# Quality of Sarcoma Care: Longitudinal Real-Time Assessment and Evidence Analytics of Quality Indicators

**DOI:** 10.3390/cancers15010047

**Published:** 2022-12-22

**Authors:** Philip Heesen, Gabriela Studer, Beata Bode, Hubi Windegger, Benjamin Staeheli, Paul Aliu, Javier Martin-Broto, Alessandro Gronchi, Jean-Yves Blay, Axel Le Cesne, Bruno Fuchs

**Affiliations:** 1 Medical Faculty, University of Zurich, 8091 Zurich, Switzerland; 2 Faculty of Medicine, University of Lucerne, 6002 Lucerne, Switzerland; 3 Swiss Sarcoma Network, 6000 Luzern, Switzerland; 4 Medical Oncology Department, University of Madrid, 28040 Madrid, Spain; 5 Department of Surgery, Fondazione IRCCS Istituto Nazionale dei Tumori, Via Venezian 1, 20133 Milano, Italy; 6 Department of Medical Oncology, Centre Léon-Bérard Lyon, 69008 Lyon, France; 7 Department of Medical Oncology, Gustave Roussy Cancer Campus, 94805 Paris, France

**Keywords:** interoperable digital platform, quality indicators, real-time assessment, value-based health care, integrated practice unit, sarcoma, data annotation

## Abstract

**Simple Summary:**

This article comprehensively defines, assesses and analyzes quality indicators of sarcoma care. A novel interoperable digital platform is presented that gathers information from physicians (work-up, therapy and MDT information) as well as patients (PROMS/PREMS) consecutively and instantly when a new event occurs, which thereby automatically provides evidence of the quality of care on all aspects. As the platform analyzes annotated real-time world information, predictive modelling and value-based health care may become a reality, thereby giving rise to precision health care in the future.

**Abstract:**

Sarcomas represent a large group of rare to very rare diseases, requiring complex management with a transdisciplinary approach. Overall progress has been hampered because of discipline, institution and network fragmentation, and there is no global data harmonization or quality standards. To report on and improve quality, a common definition of quality indicators (QIs) of sarcoma care as well as the capacity to assess longitudinal real-time data is required. An international advisory board of world-renowned sarcoma experts defined six categories of QIs, totaling more than 80 quality indicators. An interoperable (web-based) digital platform was then created combining the management of the weekly sarcoma board meeting with the sarcoma registry and incorporating patient-reported outcome measures (PROMs) into the routine follow-up care to assess the entire care cycle of the patient. The QIs were then programmed into the digital platform for real-time analysis and visualization. The definition of standardized QIs covering all physician- (diagnostics and therapeutics), patient- (PROMS/PREMS), and cost-based aspects in combination with their real-time assessment over the entire sarcoma care cycle can be realized. Standardized QIs as well as their real-time assessment and data visualization are critical to improving the quality of sarcoma care. By enabling predictive modelling and introducing VBHC, precision health care for a complex disease is on the horizon.

## 1. Introduction

Sarcomas constitute a large group of rare cancers, and their treatment is multidisciplinary and complex. There are a series of evidence- and consensus-based sarcoma guidelines available for the appropriate work-up and treatment of bone and soft tissue sarcomas. Several recent studies examined the compliance of such guidelines, as well as its positive association with clinical outcomes [1,2,3,4,5]. Due to low adherence, sarcoma treatment is preferentially regionalized to dedicated networks because specialized multidisciplinary sarcoma teams (MDT) provide improved management and care with better adherence to guidelines compared with patients who are treated outside of such networks [6,7,8,9,10,11,12]. However, regionalization of care alone does not yet guarantee quality of care, mainly because quality indicators of work-up and treatment have not yet been established at large, and a tool for their large-scale assessment with the possibility of real-life monitoring is lacking [13].

While access to an MDT- or sarcoma-integrated practice unit (IPU) care network plays a pivotal role with respect to quality care, health care costs are constantly rising, despite the fact that the increasing costs may not necessarily match the added quality [14]. Identification of the most effective ways to organize, manage, finance and deliver high-quality care (which is summarized as health services research) to reduce non-compliance to CPG and improve patient safety and outcomes will become increasingly important in the near future. Effective on April 2021, for example, Switzerland introduced a new law regarding the quality and economics of patient care, which commits all care providers to measure quality and to introduce a quality management system, which lays the foundation for value-based health care (VBHC).

VBHC was introduced more than 15 years ago by Porter et al. and is defined by the quality and outcome divided by the total costs over the entire longitudinal health care cycle [15,16,17]. It transforms a fee-for-service legacy system into a value-based system of shared values, vision, transparency and trust, thereby moving from quantity to quality and from volume to value. Meanwhile, VBHC is a widely accepted instrument to improve quality of care while reducing health care costs [18,19]. There are several important prerequisites for VBHC. First, an integral information technology platform is required, which allows the preferably real-time interoperable data exchange of IPUs over the entire globe. Second, VBHC requires the definition of quality indicators (QIs) and its assessment. Third, the established quality ultimately then has to be associated with the costs of the entire longitudinal care cycle.

A strategy for evaluating robust information on QIs is the harmonization of data by real-time prospective assessment [20,21]. This requires an interoperable digital platform, ideally between sarcoma networks and their global databases, which represents a digital mirror of prospective patient data in real time and allows the instant visualization of the data. However, it is commonly believed that within current silo hospital structures in many centers (such as data management, “added monodisciplinary” approach and currently available clinical information systems) the digital requirements widely surpass the financial preconditions to achieve such necessary data information.

As far as sarcoma treatment is concerned and to the best of the authors’ knowledge, there is no comprehensive definition of quality indicators of shared sarcoma care (beyond local-recurrence-free survival, disease-free survival, metastasis-free survival and overall survival) described in the literature. At this point, their real-time structured assessment, specifically in relation to healthcare costs, has never been realized. Herein, we provide an interoperable digital platform solution focusing on the QIs of sarcoma care.

## 2. Materials and Methods

### 2.1. Description of the Interoperable Digital Platform

SSN has established a cloud-based, transparent, harmonized, transdisciplinary, multi-institutional, prospective, real-world-time data sarcoma registry including absolute (not only sarcoma diagnosis but also all patients who undergo a biopsy for suspicion of sarcoma) patient numbers with a focus on the assessment of quality indicators (QI) of sarcoma care, covering the entire longitudinal care cycle. For this purpose, the management of the weekly multidisciplinary team (MDT)/sarcoma board (SB) meeting was coupled with the data registry. To ensure the quality and completeness of all MDT/SB decisions, every new patient and any new event or treatment change is required to be presented to the MDT/SB. Importantly, the patient has to give written consent to the collection and anonymous use of their data. For each patient presented at the MDT/SB, the treating physician of a given discipline enters the respective information. For example, the surgeon fills in the details of surgery, the radiation oncologist enters the relevant data for radiation therapy, the pathologist and radiologist enter the respective data etc. This may include some 3–5 min for a single treating physician. This is a critically important step because regular clinical information systems most often do not allow for the collection of structured data, which is pivotal for analysis. This shared and structured data collection approach assures minimal time effort and the highest possible data quality. Furthermore, because all data are discussed together at the MDT/SB and because the discussion allows open questions (such as the surgical margin) to be addressed, this meeting that includes all treating physicians is used as a collective data quality check. This setup assures the highest possible data quality in real time, including the decisions on subsequent treatments. Based on the entered data, the interoperable digital platform is programmed to calculate and automatically visualize the QIs in diagrams, tables or figures. In addition, while the MDT/SB assures data inclusion regarding all active treatments, the interoperable digital platform requests PROMS electronically from patients under follow-up and without evidence of disease at predefined intervals. This guarantees longitudinal data coverage over the entire cycle of care for all patients.

### 2.2. QI and Tools

Using a modified Delphi approach, the Swiss Sarcoma Network (SSN) international advisory board consisting of world-renowned sarcoma experts (A.L.C., J.B., A.G. and J.M.) defined holistic quality indicators of sarcoma patient care. It encompasses six categories of care aspects (Table 1), totaling more than 80 QIs (Table 2, Table 3, Table 4, Table 5, Table 6 and Table 7). To guarantee real-time data assessment over time, the patients fill out patient-reported outcome/experience measures (PROMS/PREMS) (Table 7) either at the outpatient visit or online in the case of telemedicine consultations, depending on type of treatment they had and the time point of follow-up. Overall, >385 variables are routinely assessed using the interoperable digital platform (Figure 3). The data are hosted at the Federal Institute of Technology in Switzerland (Leomed, ETH Zurich, Switzerland; https://sis.id.ethz.ch/services/confidentialrese;researchdata; accessed on 20 October 2022) to ensure the highest level of data protection with respect to interinstitutional exchange, political independence, and continuous technology developments. The interoperable digital platform allows the automated extraction of data to be used for calculations of QIs, thereby generating real-world evidence [22,23]. The digital platform allows for instant analysis and visualization, basic statistics and figure creation. The QI analysis of sarcoma care parameters can be customized according to, for example, time period, type of dignity, planned and unplanned (whoops) resections, institution, in real time and interactively. Its modular setup allows the extension and adaptation of the parameters as more data will be collected.

### 2.3. Objectives

In this study, we assessed and described the selection of Qis relating to the sarcoma work-up as well as EQ-5D (a PROM) in a four-year period using the interoperable digital platform.

## 3. Results

### 3.1. Definition of Quality Indicators of Sarcoma Care

The quality indicators of sarcoma care as defined herein encompass six categories, including the work-up of sarcoma patients, the management of the MDT/SB meeting, type of therapies, the complexity of therapy, outcome measures and PROMS/PREMS (Table 1). Each single category contains a subset of parameters (Table 2, Table 3, Table 4, Table 5, Table 6 and Table 7) that define the respective category.

### 3.2. Quality Indicators of Sarcoma Work-Up

**Figure 1:**
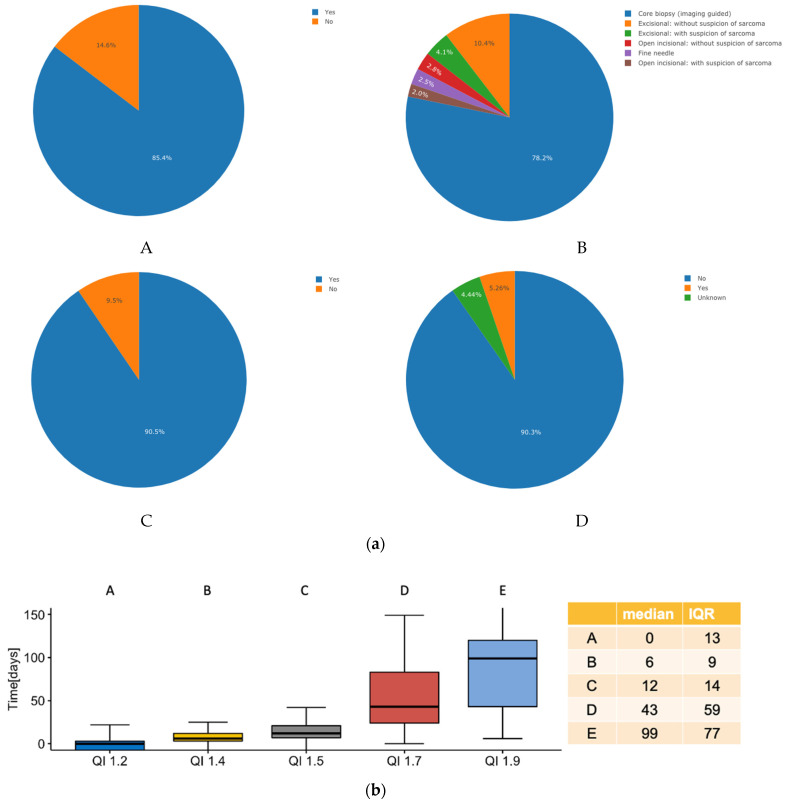
(**a**) Pie chart analysis for four of the nine quality indicators of the sarcoma work-up; (**A**), was imaging performed before biopsy? (**B**), which type of biopsy was performed? (**C**), was biopsy conducted before initiation of treatment? (**D**), was metastasis present at diagnosis? (**b**) Box plot analysis for five of the nine quality indicators of the sarcoma work-up; A, time from first patient contact to biopsy, B, time from biopsy until establishment of diagnosis, C, time from biopsy until sarcoma board presentation, D, time from diagnosis to initiation of therapy and E, time from sarcoma board presentation to initiation of treatment.

**Figure 2:**
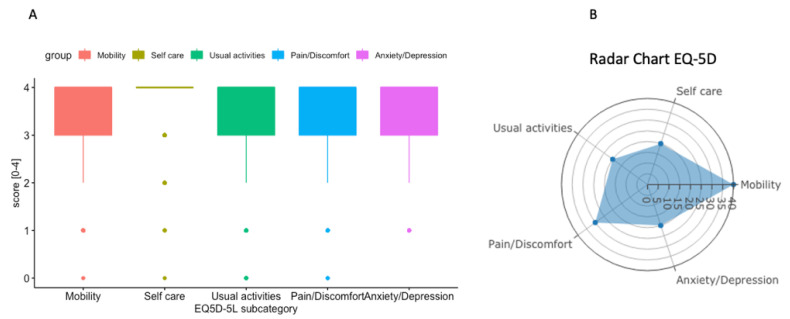
(**A**) Box plot summary of EQ-5D including 511 consecutive patients. (**B**) Radar chart summary of one single patient representing the five dimensions of EQ-5D.

The detailed results of the Qis work-up are summarized in Figure 1 and Figure 2 for 1308 patients with suspected (*n* = 719, 55%) and (*n* = 589, 45%) confirmed sarcoma presented within the SSN over a four-year time period (1 January 2018 until 31 March 2022). With respect to the QI sarcoma work-up as an example for all other quality indicators, it was observed that *n* = 1117 (85.4%) of all patients underwent radiological imaging before performing a biopsy. On the interactive website of the interoperable digital platform, this number can be instantly further analyzed and visualized according to, for example, diagnostic categories, diagnoses, anatomic regions, institution etc. to define how the respective parameter of interest may differ from the mean of the category of interest (Figure 1). This enables the discovery of strengths and weaknesses of each referral network, institution or discipline and may not only specifically define areas of improvement but also provide a benchmark for comparison with other sarcoma networks on an international level (including adjustment to the tumor characteristics and complexity of therapy). Similarly, analyzing the time from the first biopsy to first contact within the sarcoma IPU, we find a median of 0 days and an interquartile range of 13 days, which implies that half of all patients underwent the biopsy performed at the same day as the first contact or present with the biopsy, defining the efficiency of this specific part of the work-up process. With information regarding structured data on the work-up, a cost tag can be attributed to each specific step, thereby generating the effective cost for a given aspect of treatment.

### 3.3. HRQOL-EQ-5D

In the time period from the 1 October 2021 to the 31 March 2022, 511 EQ-5D questionnaires were routinely assessed in the outpatient clinic, including all patients with suspected and confirmed sarcoma, as summarized in Figure 2 [24]. These results show that the consecutive assessment of health-related quality of life as assessed by EQ-5D scores in the routine outpatient clinic is feasible. Analogous to the QIs, each subcategory can be analyzed longitudinally over time and/or by event of treatment according to clinical metrics using the interactive website. A radar chart (Figure 2B) is provided to assist the interpretation of the results of the discussion with the patient.

## 4. Discussion

Today’s competition in health care is often not aligned with shared value, and financial success of various health care stakeholders may not necessarily equal success for the patient. Because of rising health care costs and the uncontrolled growth of unstructured medical data, the creation of a novel ecosystem with data interoperability focusing on quality care evaluated from practices and patient outcomes is pivotal. The existing misalignment can be overcome with structured data collection, global data harmonization and interactive data assessment with real-time analysis to create evidence-based information.

Existing databases for sarcoma research may often underestimate the true prevalence of sarcoma. This is explained by their dependence on administrative or billing data, insufficient data coding or the use of preexisting documented hospital diagnoses codes, which are reasons why the expansion on real-world evidence-based patient-focused data sets is mandatory [20]. Furthermore, routine structured assessment and analysis of QIs play an integral role in establishing a novel ecosystem with interoperable and harmonized data exchange to improve the quality of care for sarcoma patients. The six categories of QIs span the entire cycle of care for any given disease of the patient with sarcoma suspicion and are thereby representative for the quality of sarcoma care. To the best of the authors’ knowledge, this represents an entirely novel approach that generates absolute numbers including all patients with suspicion of sarcoma (not only with confirmed sarcoma diagnosis).

Herein, we focus on two aspects of QIs to present the feasibility and routine acquisition of the setup: the QI 1 work-up and one of the PROMS (EQ-5D) (Table 1). The QI sarcoma work-up consists of eight questions, which cover the key aspects of preparing a patient for treatment. Detailing the work-up is important, particularly with respect to the historically unchanged and unacceptably high rate of unplanned (“whoops“) resections, where more precise data may foster the understanding of why these still occur today. Additionally, the QI 1 work-up includes a second review by an expert sarcoma pathologist, which is a critical and established quality indicator, but it still does not represent common practice mainly because the structure of many current databases simply does not automatically generate such information. Obviously, the definitions always remain debatable but can easily be expanded by further aspects if sarcoma experts deem it necessary. For this purpose, the sarcoma academy (www.sarcoma.academy; accessed 20 October 2022) was founded to enable inter-disciplinarily exchange of cases and to discuss and update the QIs on the global level. The analysis of quality indicators as presented herein summarizes the routine assessment of 1308 patients over a four-year period of one single MDT/SB network and may provide a benchmark for future national and international comparisons.

HRQoL questionnaires are increasingly recognized as a pivotal tool for reporting outcome measures in the medical practice [18,19]. In oncology specifically, the EORTC-QLQ-C30 and the EQ-5D are widely recognized and were introduced for sarcoma patients years ago [25,26]. However, their routine clinical use for sarcoma patients in daily practice has not yet been shown, and there is also not a reference score for all sarcoma entities and respective anatomic locations available. In the current study, our digital platform allows the consecutive assessment of all outpatient visits in daily routine practice. We currently assess the EQ-5D at each single clinical visit, and the EORTC-QLQ-C30 is assessed in addition for all patients who underwent chemotherapy. Overall, the six QI categories cover the entire cycle of patient care, which is further detailed by the single aspects. This setup allows the identification of weak and strong areas, thereby facilitating improvement in sarcoma care within an MDT/SB. It further allows benchmarking and comparison of treatment quality among various MDT/SB in distant geographic areas, which directly benefits the patient. As such, a global definition and comparison of sarcoma shared care becomes possible, which enables us to define areas of improvement based on harmonized data and evidence analysis. Importantly, through global harmonization of data quality, transdisciplinary and structured data can be assessed independent of the location and become interchangeable, and multi-institutional international collaboration on a rare disease is greatly facilitated. It is envisioned that herewith, a sarcoma quality score for a respective MDT/SB network (the center itself and its associated regional network) can be defined and introduced for international benchmark comparisons while importantly respecting the complexities associated with treatment. This may greatly impact the quality of international trials through the generation of more robust data and improved care quality for the patient. Having information regarding structured data on the entire cycle of care including the work-up enables a cost tag to be attributed to each specific step, which thereby generates an effective cost for a given aspect of treatment.

**Figure 3:**
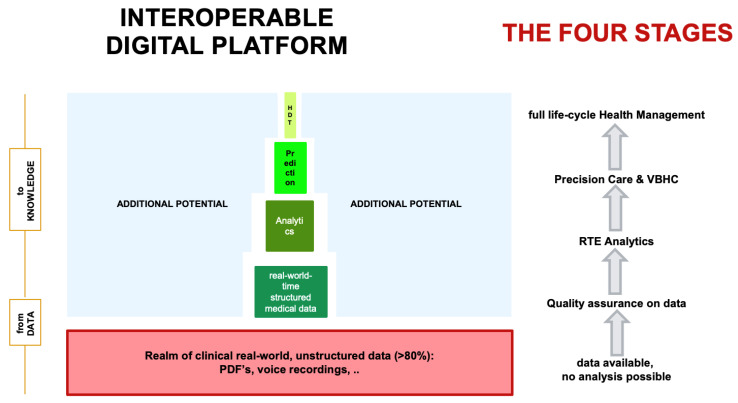
The interoperable digital platform is designed to receive longitudinal, absolute, routine, structured, real-world-time data from daily clinical routine practice. The MDT/SB fulfills the purpose of data quality control. Analytics of these data generate real-time evidence, which is used for precision modelling (through artificial intelligence and machine learning approaches) as well as value-based health care (through attribution of cost tags with structured data). Abbreviations. RTE, real-time evidence; VBHC, value-based health care; HDT, human digital twin.

Cost increases in health care is a universal problem. Porter et al. introduced the concept of value-based health care (VBHC) [16,17,27]. Herein, shared value is defined by the quality of patient care and outcome divided by the total cost over the entire cycle of care [28,29]. The prerequisite to introduce VBHC to create a new ecosystem is the definition of quality. Because the digital interoperable platform encompasses the entire cycle of care and because each structured QI parameter can be attributed a cost tag, it will become possible for the first time to determine the entire costs of the treatment for a given disease and patient (Figure 3). Above all, cost containment will be based uniquely on quality measures with such a setup, which enables the introduction of VBHC through disruption of the current ecosystem. Importantly, this has great potential to achieve cost containment for the benefit of the patient as well as a sustainable health care system [30].

Data are considered the key driver for the evolution from one-size-fits-all to precision medicine [21,31]. Predicting outcomes has been recognized as a major focus in today’s health care, and currently, nomograms are still routinely used in daily sarcoma practice [32,33]. However, these are most often based on retrospective data, sometimes even more than 20 years old, without harmonized data acquisition and definitions and with all institutions independent from each other. Although validation has been performed, calibration curves imply wide ranges of survival predictions and therefore may imperfectly mirror reality. Therefore, because the technical infrastructure is now able to integrate a common, internationally defined and harmonized data language into daily practice, real-time prospective data acquisition may be regarded as today’s standard and daily routine. Focusing data acquisition on quality standards, the patient can transparently monitor the quality of the treatment according to international guidelines and quality standards on his own. Having such structured quality data available at a large scale, predictive outcome analytics to individualize treatment decisions based on outcome prediction becomes a reality (Figure 3). As of now, we have integrated clinical data (to represent physician-based data), PROMS & PREMS (to represent patient-based data) as well as data of the costs of the work-up and treatment of patients (to represent health care cost). In the future, we will integrate multi-omic (genomics, epigenomics, proteomics, metabolomics, etc.) functional tumor profiling data for clinical decision support such as realized with the TumorProfiler® or through semantic web technologies [34]. The combination of clinical data and bioinformatics data allow predictive outcome analytics as a standard of care in the very near future, and digital twin computing (DTC) becomes reality.

Obviously, the exchange and security of patient data remains a constant challenge for global collaboration, as the different laws on data privacy and security issues are usually country specific. However, and because the assessment of quality of sarcoma care—which is in strong contrast to Alphabet, Amazon or Alibaba—focuses not on the individual patient’s data but is aggregated, the exchange of anonymized data with the respective patient’s consent such as is widely practiced by pharma companies is becoming a reality. Furthermore, one may argue that extrapolating on health care data from a small country with an intricate network of small hospitals in semi-autonomic political areas within close distances and a BIP of close to 12% may not be representative for other countries with different geographies and demographies. However, as previously mentioned, the QIs were determined by sarcoma experts from different countries and from the view of the patient, which is independent of where health care is requested and is therefore considered similar worldwide. Furthermore, the presented QIs may not be considered comprehensive. The system is set up such that any new parameter may be modularly integrated into the interoperable digital platform.

## 5. Conclusions

It has been recognized for decades that the most pressing questions can only be answered through international collaboration. This has, however, not yet materialized due to the lack of means and technology. The interoperable digital platform takes advantage of current technological opportunities, which defines a common language focused on the quality of sarcoma care, allows real-time assessment over the entire care cycle, strengthens the collaboration of IPUs and transparently integrates and visualizes quality analytics to realize an international exchange. It may ultimately pave the way to realizing precision medicine through predictive modelling and introducing VBHC for a complex disease, which may serve as a role model for other diseases.

## Figures and Tables

**Table 1:** Overview of groups of quality indicators.

QI FOR MULTIDISCIPLINARY TEAMS (MDT)
1. Sarcoma work-up of patients
2. MDT/SB management
3. Therapy (incl. surgery, radiation-, chemotherapy)
4. Complexity of sarcoma therapy
5. Clinical metrics outcome (physician based)
6. PROMS/PREMS (patient based)

**Table 2:** Sarcoma work-up.

QUALITY INDICATORS: SARCOMA WORK-UP
• was imaging performed before biopsy
• time from first patient contact to biopsy
• which type of biopsy was performed
• time from biopsy to MDT/SB presentation
• time from biopsy to SB presentation
• was biopsy performed before initiation of treatment? separated according to type of treatment
• was there metastasis at presentation
• time from MDT/SB presentation to initiation of treatment (incl. analysis depending on type of therapy

**Table 3:** MDT/SB management.

QUALITY INDICATORS: MDT/SB MANAGEMENT
• how many patients were presented per month/per year
• how many presentations took place per month/per year
• how many first presentations
• how many follow-up presentations
• how many bone lesions—superficial/deep soft tissue lesions were presented
• how many malignant—intermediate—benign lesions were discussed
• how many decisions on: - surgery - radiation therapy - combination radiation therapy–surgery - combination chemotherapy–surgery - combination surgery–radiation therapy–chemotherapy
• how many decisions were realized/executed? - Overall - surgery - radiation oncology - chemotherapy
• how many patients were presented over entire cycle of care

**Table 4:** Therapy.

QUALITY INDICATORS: THERAPY
• % margin status (R0, R1, R2) at definitive surgery • surgical, pathological, consens
• % amputations
• % preoperative radiation therapy (yes/no)
• % postoperative radiation therapy (yes/no)
• % neoadjuvant chemotherapy (yes/no)
• % adjuvant chemotherapy (yes/no)

**Table 5:** Complexity of therapy.

QUALITY INDICATORS: COMPLEXITY OF THERAPY	
• surgical complexity STS	Cancers March 2022
• surgical complexity bone sarcoma	Age, grading/type of lesion, prior RT, chemo/whoops, size of lesion, location, resected structures, reconstructed structures, involved disciplines
• surgical complexity visceral sarcoma	Age, grading/type of lesion, prior RT, chemo/whoops, size of lesion, location, resected structures, reconstructed structures, involved disciplines
• radiation oncology complexity treatment	Aim of RT (curative, locally curative, palliative, definitive, unknown); RT technique (IMRT, VMAT, SRT, 3DCRT, 2DCRT, unknown; RT type (photons, protons, electrons, brachytherapy (transient, permanent), conventional, other, unknown); total dose/number of fractions; GTV/PTV; Grade III/IV toxicities;
• systemic treatment complexity	Aim of systemic therapy (curative intent pre/postop, additive, maintenance, palliative); number of curative/palliative cycles planned/executed; time to next treatment (TTT); reasons for discontinuation (completed, discontinued (toxicity, PD, planned, patient‘s wish, death); Grade III/IV toxicities

**Table 6:** Outcome.

QUALITY INDICATORS: OUTCOME
• local recurrence within 1st year after tumor resection
• local recurrence overall
• systemic recurrence with 1st year of treatment initiation
• systemic recurrence overall
• latest follow-up: NED, AWD, DOD, DOR, no assessment possible; lost to followup, unknown)
• in case of RT: % vascular disorders (lymphedema, ROM, fibrosis); skin disorders (hyper-,hypopigmentation); bone disorders (osteonecrosis)
• in case of chemotherapy: % therapy during last 3 months of life.

NED, no evidence of disease; AWD, alive with evidence of disease; DOD, dead of disease; DOR, death of other reasons.

**Table 7:** PROMS/PREMS.

SARCOMA QUALITY INDICATORS	PROMS/PREMS
• work-up/regular f-up	-WHO-ECOG -EQ-5D -EQ-VAS -work ability index
• biopsy	-biopsy
• surgery	-MSTS upper/lower extremity -TESS upper/lower extremity -visceral
• radiation oncology	-local effects of RT
• chemotherapy	-EORTC-QLQ-C30 -MDASI
• therapy focused	-cancer therapy satisfaction -satisfaction with RT -control preferences
• Physican related	-CARE
• Institution focused	-satisfaction with institution

## Data Availability

https://www.swiss-sarcoma.net/ (accessed on 22 November 2022).
